# Mesenchymal stem cell transplantation can restore lupus disease-associated miRNA expression and Th1/Th2 ratios in a murine model of SLE

**DOI:** 10.1038/srep38237

**Published:** 2016-12-07

**Authors:** Eun Wha Choi, MinJae Lee, Ji Woo Song, Il Seob Shin, Sung Joo Kim

**Affiliations:** 1 Laboratory Animal Research Center, Samsung Biomedical Research Institute, 81 Irwon-ro, Gangnam-gu, Seoul 135-710, Republic of Korea; 2 School of Medicine, Sungkyunkwan University, 81 Irwon-ro, Gangnam-gu, Seoul 135-710, Republic of Korea; 3 Biostar Stem Cell Research Center, K-STEMCELL, #2-305 IT Castle, 98 GasanDigital2-ro, Geumcheon-gu, Seoul 153-768, Republic of Korea; 4 Department of Surgery, Division of Transplantation, Samsung Medical Center, Sungkyunkwan University School of Medicine, 81 Irwon-ro, Gangnam-gu, Seoul 135-710, Republic of Korea

## Abstract

C3.MRL-Fas^lpr^/J mice spontaneously develop high titers of anti-dsDNA, mild glomerular nephritis, and severe lymphoproliferation symptoms. This study aimed to compare the effects of long-term serial administration of human adipose tissue-derived mesenchymal stem cells (ASCs), and cyclophosphamide treatment in C3.MRL-Fas^lpr^/J mice using a murine SLE model. C3.MRL-Fas^lpr^/J mice were divided into saline (C), cyclophosphamide (Y), and ASC (H) treatment groups. Background-matched control C3H mice treated with saline (N) were also compared. The Y group showed the greatest improvement in disease parameters, but with damaged trabecular integrity. ASC transplantation reduced anti-dsDNA levels, glomerular C3 deposition and CD138 proportion significantly, without trabecular damage. Furthermore, both cyclophosphamide and ASC treatment significantly decreased the ratio of Th1/Th2 compared with the saline-treatment. The expression levels of miR-31-5p, miR-96-5p, miR-182-5p, miR-183-5p, and miR-379-5p were significantly higher, while those of miR150-5p were significantly lower in the C group than in the N group. The expression levels of miR-96-5p, miR-182-5p in the Y and H groups were significantly lower than in the C group. Thus, treatment with cyclophosphamide or ASC can change miRNAs and decrease miR-96-5p and miR-182-5p expression, as well as decreasing the CD138 proportion and the Th1/Th2 ratio, which might be involved in the therapeutic mechanism.

Systemic lupus erythematosus (SLE) is a chronic multisystemic autoimmune disease caused by interplays between genetic factors, inappropriate immune regulation, and other factors, such as hormonal and environmental variables. The contribution of epigenetic regulatory defects, including abnormal DNA methylation, histone modification, and miRNA regulation, to lupus pathogenesis also has been suggested^1^,^2^,^3^.

Several murine models of SLE have been used to understand its pathogenesis and to evaluate the efficacy of SLE therapeutics^4^,^5^. MRL mice homozygous for lymphoproliferation spontaneous mutation, *Fas*^*lpr*^ (MRL/MP- *Fas*^*lpr*^*/Fas*^*lpr*^, MRL/lpr mice) develop a human lupus-like syndrome that includes glomerulonephritis, vasculitis, organ weight gain and autoantibody production^6^,^7^. *Fas*^*lpr*^ (*lpr*) has an early transposable element inserted into intron 2 of *Fas*; hence, it expresses low levels of Fas. MRL/MPJ mice develop autoantibodies, late-onset chronic nephritis and die at 2 years of age, and the *lpr* mutation of the *Fas* gene accelerates the onset of autoimmune disease. Thus, MRL/lpr mice develop severe early-onset autoimmune disease and severe glomerulonephritis. According to the Jackson’s strain information (https://www.jax.org/strain/000485), female MRL/lpr mice die at an average age of 17 weeks and males at 22 weeks. The *lpr* gene can be transferred to genetically distinct strains by a series of cross-intercross mating and the phenotype and severity of autoimmune manifestations induced by the *lpr* gene varies considerably between mice of different strain backgrounds^8^,^9^. C3H/HeJ background *Fas*^*lpr*^*/Fas*^*lpr*^ (C3.MRL-Fas^lpr^/J) female mice die at an average age of 42–52 weeks and develop negligible glomerulonephritis^9^,^10^.

Aberrant miRNA expression patterns have been evident in various pathologic conditions^11^, and miRNAs play a critical role in the regulation of immune cell development and immune responses, and in the maintenance of immune homeostasis^12^. The involvement of miRNAs in immune tolerance control and autoimmunity has also been reported^13^. Dai *et al*. reported that a common lupus disease-associated miRNA expression pattern is present in splenocytes in three different murine models of lupus^14^. Despite the genetic differences among the three murine lupus models (MRL-lpr, B6-lpr and NZB/W F1), miR-182-96-183 cluster, miR-31 and miR-155 were markedly upregulated in splenocytes when compared with age-matched control mice^14^.

The development of a new strategy and trials of alternative therapies are required to resolve these serious toxicities, and there is no uniform efficacy of currently used drugs, such as immunosuppressive drugs and corticosteroids, in SLE^15^. Mesenchymal stem cells (MSCs) are attractive, focused therapeutic tools for the treatment of various diseases, including autoimmune diseases, due to their many advantageous properties, such as their capacity for differentiation, transdifferentiation, homing activity, and immunomodulatory and protective effects by paracrine factors, such as trophic, chemoattractant, anti-scarring and immunomodulatory factors^16^,^17^. Adipose tissue-derived MSCs (ASCs) are considered an ideal source of these stem cells because of their plentiful supply, availability, non-immunogenic properties, and minimal ethical considerations^18^,^19^. Furthermore, their capacity for proliferation and differentiation is less likely to be affected by aging relative to bone marrow-derived MSCs, as evaluated by telomerase activity, p21 gene expression, and senescence-associated β-galactosidase activity^20^.

Our previous studies revealed that ASC transplantation significantly improved serologic, immunologic, and histologic abnormalities, decreased the incidence of proteinuria, and increased the survival rate in NZB/W F1 mice^21^,^22^, and also prevented the development of lupus dermatitis in MRL-lpr mice^23^.

Patients with SLE demonstrate a wide range of symptoms and severities. A difficulty in the translational study of SLE is that one certain preclinical model cannot recapitulate the human SLE condition. For example, MRL/lpr mice demonstrate relatively severe clinical signs of human SLE, whereas C3.MRL-Faslpr/J mice present mild symptoms. Therefore, it is desirable to evaluate the clinical responses of therapies in multiple models.

The purpose of this study was to evaluate the effect of long-term serial ASC in a mild SLE model in C3.MRL-Fas^lpr^/J mice. We also intended to examine whether immunosuppressant or ASC treatment can modify lupus disease-associated miRNAs; we compared the changes of common lupus disease-associated miRNA expression patterns in C3.MRL-Fas^lpr^/J mice (mild SLE phenotype) and MRL/lpr mice (severe SLE phenotype) after long-term serial ASC (same origin) or cyclophosphamide treatments.

## Results

### Cyclophosphamide treatment significantly reduced lymphadenopathy and splenomegaly, but ASC treatment did not

**Figure 1:**
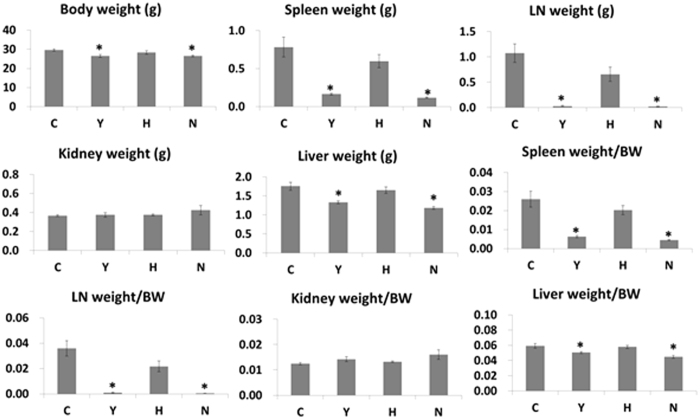
Body weight and organ weights in C3.MRL-Fas^lpr^/J mice and background matched control C3H mice after treatment. The mean weights of the spleen, lymph node, and liver, as well as individual organ/body weight ratios are presented. C: control group (C3.MRL-Fas^lpr^/J mice), Y: cyclophosphamide-treatment group (C3.MRL-Fas^lpr^/J mice), H: ASC-treatment group (C3.MRL-Fas^lpr^/J mice) and N: normal group (Background-matched control C3H mice).

The *lpr* mutation of the *Fas* gene show nonmalignant lymphadenopathy and splenomegaly associated with a characteristic expansion of autoreactive lymphocytes. Thus, we measured body weight and organ weight and calculated the organ weight:body weight ratio in order to investigate whether long-term serial ASC or cyclophosphamide treatment reduces lymphadenopathy and splenomegaly. The mean weights of the spleen, lymph node, and liver, as well as individual organ/body weight ratios were lower in all treatment groups than in the C group, and a significant difference in those weights and ratios was found in the Y and N groups, compared with the C group (Fig. 1).

### ASC or cyclophosphamide treatment increased survival rates and significantly decreased the level of anti-dsDNA antibodies

At the end of the study (at 40 weeks of age), 86.7% (13 of 15 mice), 100%, 100%, and 100% of the C, Y, H, and N groups, respectively, were still alive. Two mice in the control group that died exhibited mild and mild-to-moderate glomerulonephritis, respectively. One mouse was considered to have proteinuria; urine protein concentrations were 144.8 mg/dl and 144.5 mg/dl in last two consecutive tests before death and urine creatinine concentrations were 110 mg/dl and 50 mg/dl in last two tests before death.

**Figure 2:**
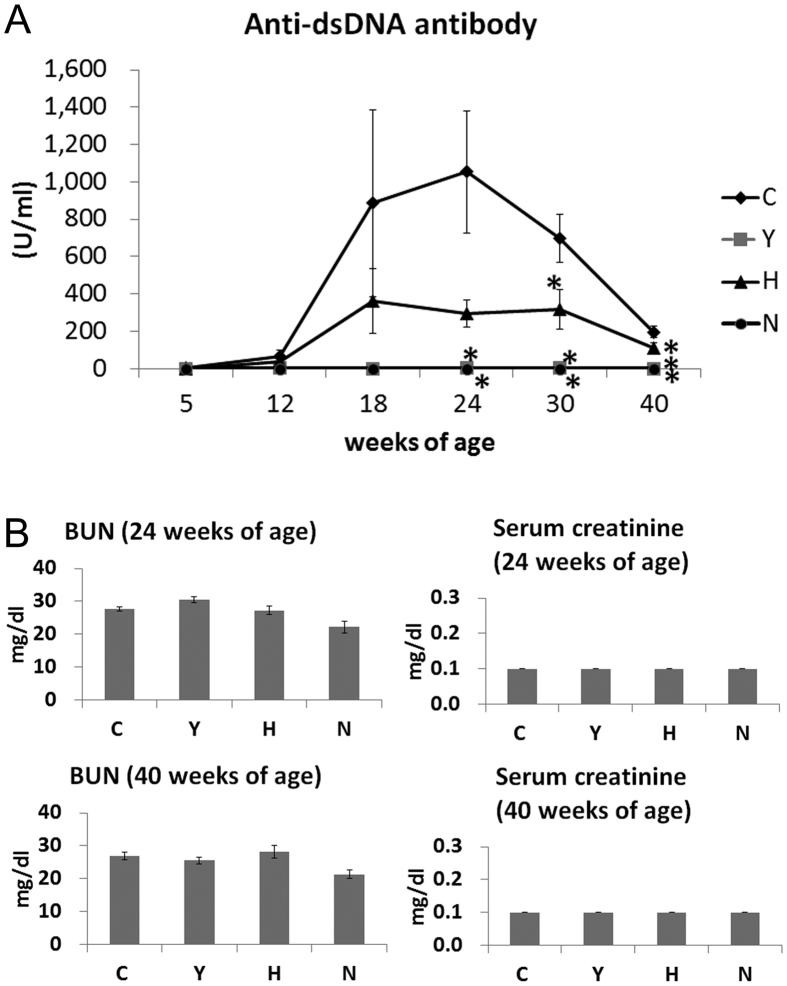
Anti-double-stranded DNA antibody, BUN and serum creatinine levels in C3.MRL-Fas^lpr^/J mice and background matched control C3H mice after treatment. (**A**) Anti-double-stranded DNA antibody (anti-dsDNA Ab). (**B**) BUN and serum creatinine concentration. Data obtained from each group were compared using a one-way analysis of variance (ANOVA) followed by *post hoc* Tukey’s multiple comparison tests. *significant (*p* < 0.05) differences from the control (**C**) group are indicated by an asterisk. N: normal group (C3H), C: control group (C3.MRL-Faslpr/J), saline-, Y: cyclophosphamide-, H: ASC-treatment group.

To investigate the level of autoantibodies and kidney function, we measured BUN, serum creatinine, and anti-dsDNA antibodies. The levels of anti-dsDNA antibodies in the Y, H and N groups were significantly lower than those in the C group in sera collected from mice at 24, 30, and 40 weeks of age (ANOVA followed by *post hoc* Tukey’s multiple comparison tests, Fig. 2A).

The urine protein concentrations of the C, Y, H and N groups at 32 and 40 weeks of age are presented in Fig. 1B. The urine protein/creatinine ratios (UP/C) of those groups at 32 and 40 weeks of age are also presented in Supplementary Fig. 1.

The incidence of severe proteinuria (>300 mg/dL) in the C group at 40 weeks of age was 7.7%; among the other groups, the incidence was 0%. The concentration of serum creatinine was not significantly different among the groups. The mean concentrations of BUN from each group were within reference values and were not significantly different among the C, Y and H groups (Fig. 2B).

### ASC or cyclophosphamide treatment decreased the proportion of CD138 cells and the ratio of Th1/Th2

**Figure 3:**
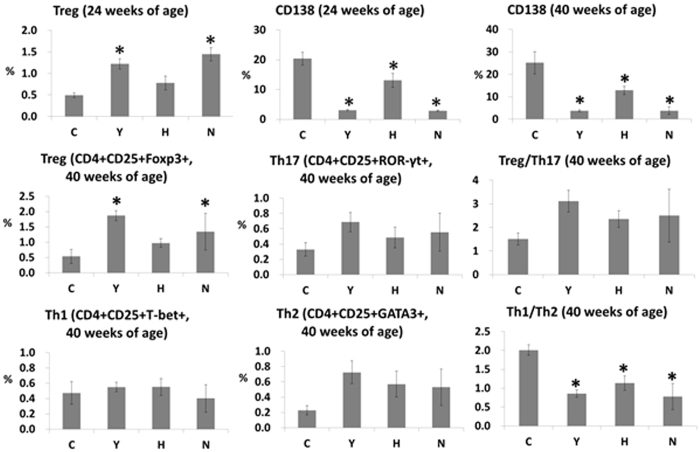
Flow cytometry data in C3.MRL-Fas^lpr^/J mice and background matched control C3H mice after treatment. N: normal group (C3H), C: control group (C3.MRL-Faslpr/J), saline-, Y: cyclophosphamide-, H: ASC-treatment group. Data obtained from groups were analyzed using one-way analysis of variance followed by *post hoc* Tukey’s multiple comparison tests. *significant (p < 0.05) differences from the control (**C**) group are indicated by an asterisk.

To determine whether ASC or cyclophosphamide treatment alters spleen cell populations, spleen cells were analyzed to estimate the proportion of Treg, Th17, Th1, Th2, and CD138 cells. The proportion of CD4+ CD25+ FoxP3+ cells in the Y and N groups was significantly higher than in the C group. Spleen cells in the H group showed a similar tendency, although this failed to reach statistical significance. The proportions of CD138 cells and the ratios of Th1/Th2 were significantly lower in the Y, H and N groups than in the C group (Fig. 3). A representative gating scheme and representative dot plots are presented in Supplementary Fig. 2.

### ASC or cyclophosphamide treatment restored the expression levels of miR-96-5p and miR-182-5p in C3.MRL-Fas^lpr^/J mice, and the expression levels of miR-96-5p and miR-182-5p showed strong positive correlations with some SLE parameters

**Figure 4:**
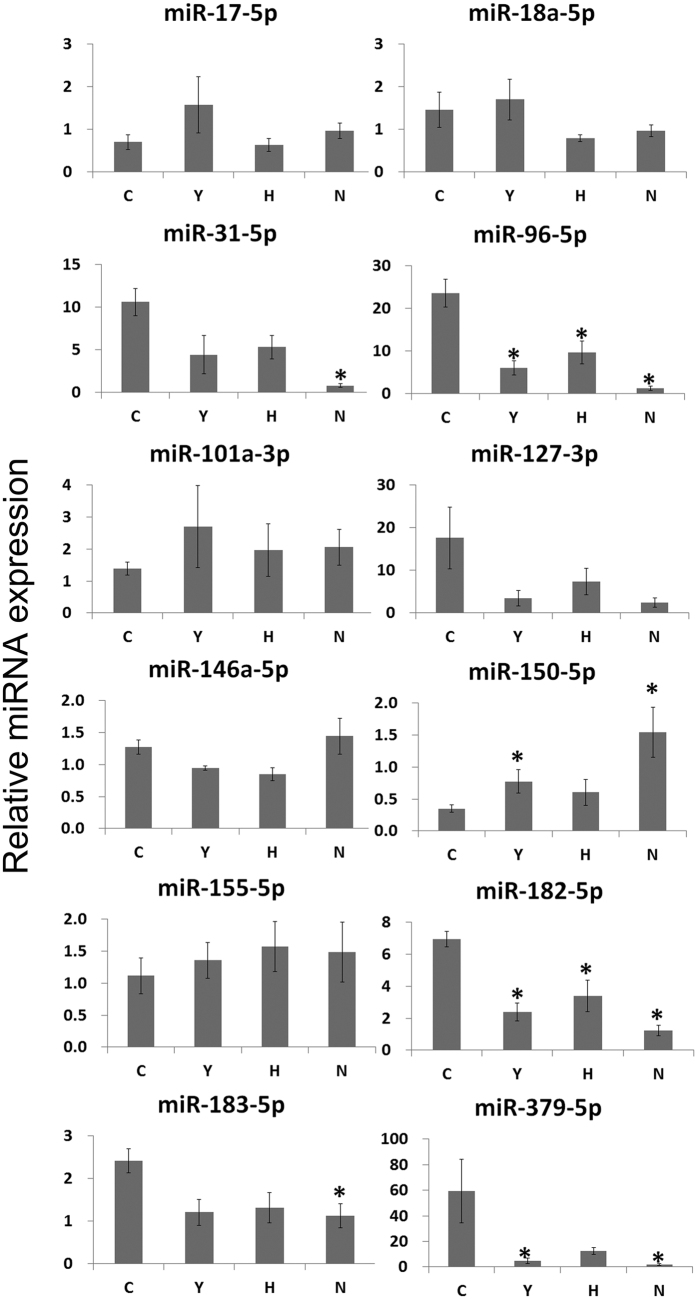
Comparison of the expression levels of common lupus disease-associated miRNAs in spleens among the groups. The expression levels of common lupus disease-associated miRNAs in spleens. Data are normalized to those of RNU6-2 and SNORD96A (average CT) and are expressed as mean (bars) ± SEM (error bars) of the relative amount compared to one sample from the normal group. Data obtained from groups were analyzed using a one-way analysis of variance followed by *post hoc* Tukey’s multiple comparison tests. *significant (p < 0.05) differences from the control (C) group are indicated by an asterisk. N: normal group (C3H), C: control group (C3.MRL-Faslpr/J), saline-, Y: cyclophosphamide-, H: ASC-treatment group.

To examine whether ASC or cyclophosphamide treatment can modify disease-associated miRNAs, we compared the expression levels of the common lupus disease-associated miRNAs between the different treatment groups. The expression levels of miR-31-5p, miR-96-5p, miR-182-5p, miR-183-5p, and miR-379-5p were significantly higher, while those of miR150-5p were significantly lower in the C group than in the N group. The expression levels of miR-96-5p, miR-182-5p, and miR-379-5p were significantly lower, while those of miR150-5p were significantly higher in the Y group than in the C group. The expression levels of miR-96-5p, miR-182-5p in the H group were significantly lower than in the C group (Fig. 4).

In splenocytes from the MRL-lpr mice (the samples in our previous study), the expression levels of miR-18a-5p, miR-31-5p, miR-96-5p, miR-127-3p, miR-182-5p, miR-183-5p, and miR-379-5p were significantly higher, while those of miR-101a-3p and miR150-5p were significantly lower in the C group than in the N group. The expression levels of miR-31-5p, miR-96-5p, miR-127-3p, miR-182-5p, miR-183-5p, and miR-379-5p 5p in the Y group were significantly lower than in the C group (Supplementary Fig. 3).

Relationships between miR-96-5p expression level and the other SLE parameters analyzed in this study were examined. The expression level of miR-96-5p showed strong positive correlations (r > 0.7) with SLE parameters of miR-182-5p, spleen weight, lymph node weight, spleen weight/body weight, lymph node weight/body weight, glomerular C3 deposition, anti-dsDNA antibody levels (at 40 weeks of age), percentage of CD138 + cells, percentage of T-bet+ of CD4+ CD25+ cells, and Th1/Th2. The expression level of miR-96-5p showed a strong negative correlation (r < −0.7) with percentage of CD4+ CD25+ Foxp3+ cells (Pearson’s correlation, Table 1).

Relationships between miR-182-5p expression level and the other SLE parameters analyzed in this study were also examined. The expression level of miR-182-5p showed strong positive correlations with SLE parameters of miR-96-5p, spleen weight, lymph node weight, spleen weight/body weight, glomerular C3 deposition, percentage of CD138+ cells, percentage of T-bet+ of CD4+ CD25+ cells, and Th1/Th2. The expression level of miR-182-5p also showed a strong negative correlation with percentage of CD4+ CD25+ Foxp3+ cells (Pearson’s correlation, Table 1).

### ASC or cyclophosphamide treatment prevented inflammatory cell infiltration in the kidney and decreased C3 deposition in the glomerulus

**Figure 5:**
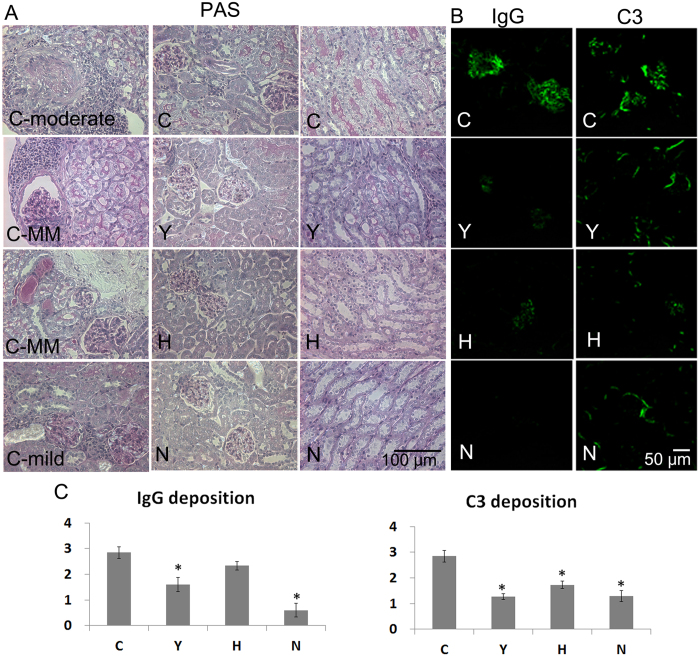
Histological staining and immunofluorescence of kidney samples. (**A**) Periodic acid-Schiff staining of kidney tissue. Bar = 50 μm. (**B**) Immunofluorescent staining of kidney samples from the experimental groups was conducted using FITC-anti IgG or FITC-anti C3 antibodies to compare the effects of various treatments. Bar = 50 μm. (**C**) IgG and C3 deposition scores in the glomerula. The intensity of fluorescence was graded on a scale of 0 (none) to 4 (great fluorescence intensity). Data obtained from each group were compared using ANOVA followed by *post hoc* Tukey’s multiple comparison tests (C: n = 13, Y: n = 15, H: n = 15, and N: n = 10). *significant (*p* < 0.05) differences from the control (**C**) group are indicated by an asterisk. MM: mild to moderate, N: normal group (C3H), C: control group (C3.MRL-Faslpr/J), saline-, Y: cyclophosphamide-, H: ASC-treatment group.

Upon histopathological examination of the kidney, one of thirteen mice (7.7%) in the C group showed moderate glomerulonephritis with enlarged glomeruli and focal crescent formation. Three of thirteen mice (23.1%) in the C group showed mild-to-moderate glomerular change and mild infiltration of inflammatory cells in the interstitium and surrounding vessels. Four of 13 mice in the C group (30.8%), three of 15 mice in the Y group (20%), three of 15 mice in the H group (20%) and one of 10 mice (10%) in the N group exhibited mesangial hypercellularity in a small percentage of glomeruli and a small number of inflammatory cells in the interstitium and surrounding vessels. Other mice showed just a small number of inflammatory cells; the C group exhibited more inflammatory cell infiltration than other groups (Fig. 5A). The fluorescence intensity of IgG deposition in the Y and N groups was significantly lower than in the C group. The fluorescence intensity of C3 deposition in the Y, H and N groups was significantly lower than that in the C group (Fig. 5B,C).

### ASC treatment did not change serum cytokine levels in C3.MRL-Faslpr/J mice

The levels of IL-4, IL-10, IL-1β, and TNF-α were significantly different among the groups (Kruskal-Wallis). To compare the cytokine levels of each treatment group versus the control group, the Mann-Whitney U test was used; the levels of these cytokines were significantly different between the C group and the Y group and were also significantly different between the C group and the N group (Mann-Whitney U test, Supplementary Fig. 4). The mean levels of IL-4 and IL-10 in sera from the H group were the highest, and those of IL-1α, IL-1β, and TNF-α from the C group were the highest. But the levels of all cytokines were not statistically significant between the C group and the H group (Mann-Whitney U test).

### Cyclophosphamide treatment had a negative effect on trabecular bone integrity

**Figure 6:**
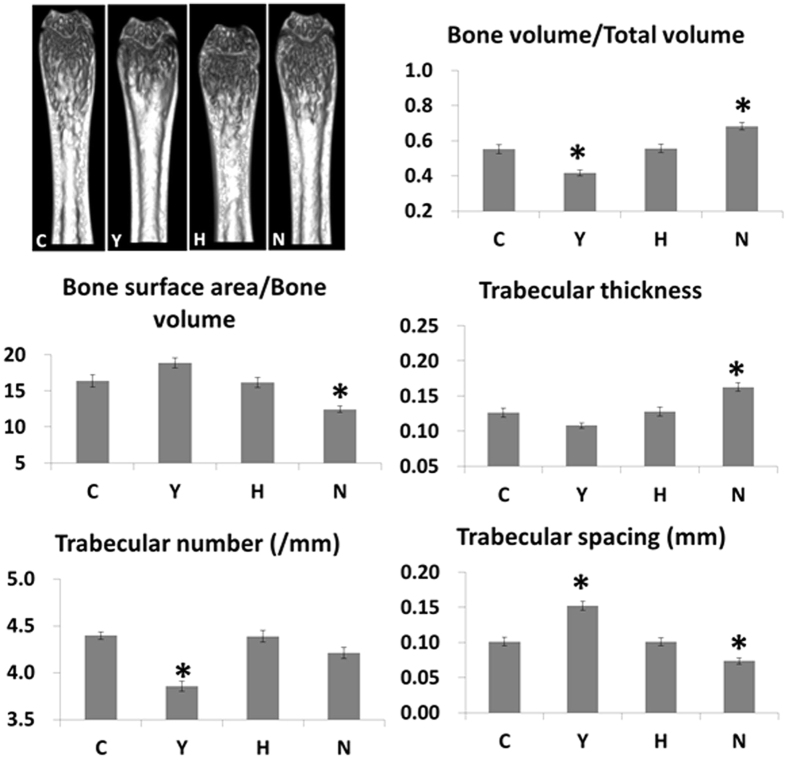
Micro-CT images from the distal femoral metaphysis and bone morphometric analyses. Representative micro-CT images of the distal femoral metaphysis and the bone volume relative to tissue volume, bone surface area relative to bone volume, trabecular thickness, trabecular number and trabecular spacing values from the experimental groups. Data obtained from each group were compared using ANOVA followed by *post hoc* Tukey’s multiple comparison tests (C: n = 13, Y: n = 15, H: n = 15, and N: n = 10). *significant (*p* < 0.05) differences from the control (C) group are indicated by an asterisk. N: normal group (C3H), C: control group (C3.MRL-Faslpr/J), saline-, Y: cyclophosphamide-, H: ASC-treatment group, Bone volume relative to tissue volume (BV/TV, C), trabecular number (Tb.N, D), and bone surface area (BS, E).

To verify the osteoporotic changes in the skeletal structures of mice, femurs (the distal femoral metaphysis) were removed from all mice and examined using micro-CT, because osteoporosis is commonly reported in SLE. The bone volume/total volume and trabecular number in the Y group were significantly lower than in all other groups, and the trabecular spacing in the Y group was significantly greater than in all other groups (Fig. 6). Thus, cyclophosphamide-treatment had a negative effect on trabecular bone integrity.

### Biodistribution of ASC

**Figure 7:**
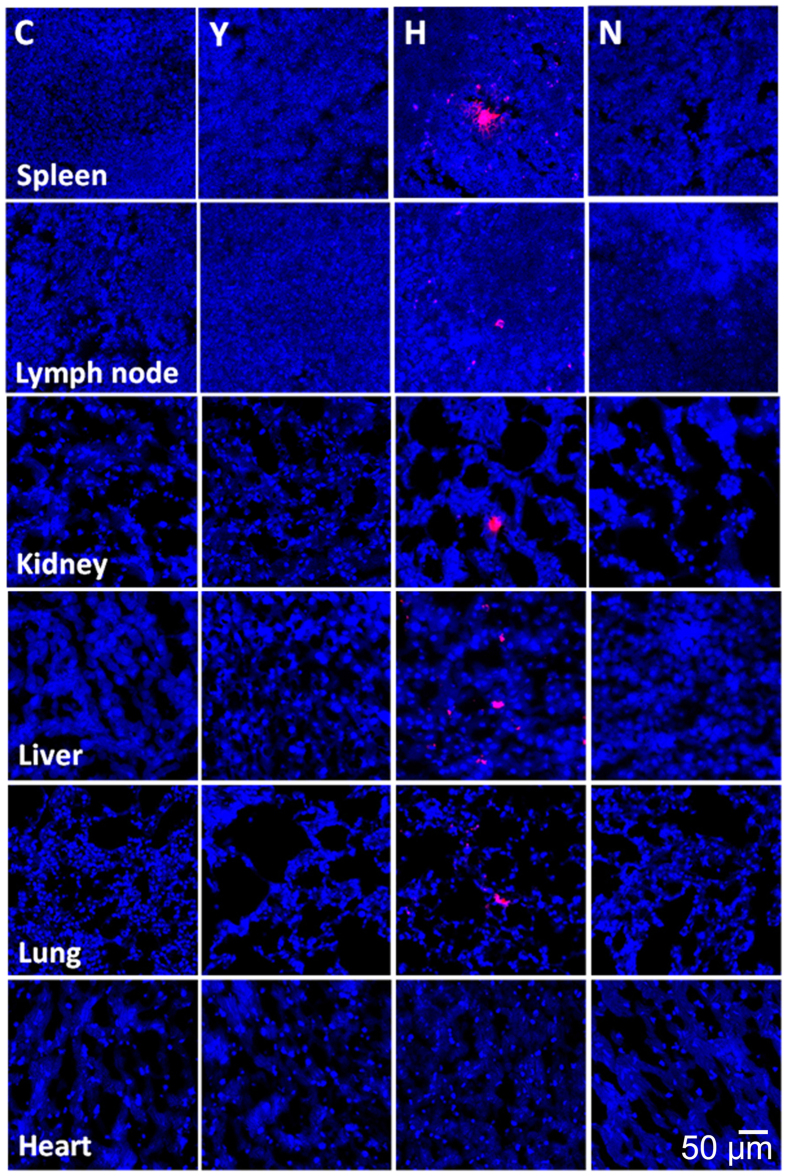
Biodistribution of transplanted adipose tissue-derived mesenchymal stem cells (ASCs). Three mice from the H group were administered ASCs fluorescently labeled with conjugated red fluorochrome Cell Tracker CM-DiI to allow identification in histopathological sections. The tissue samples were harvested at the end of the study (40 weeks of age). The presence of CM-DiI-labeled cells was examined in various tissues, such as the spleen, lymph node, kidney, liver, lung and heart (by counterstaining with mounting medium containing DAPI), with a laser scanning confocal microscope. Representative figures from each group are presented. Bar = 50 μm.

ASCs labeled with the CM-DiI red fluorescent tracker dye were present in the spleen, lymph node, kidney, liver and lung. However, little evidence of cell fluorescence was found in the heart (Fig. 7).

## Discussion

Long-term serial ASC treatment did not cure lymphoid hyperplasia in the C3.MRL-*Fas*^*lpr*^/J mice as it did in the MRL/lpr mice. In contrast, cyclophosphamide treatment decreased the spleen and lymph node weight significantly compared with the saline-treatment. The magnitude of lymphoid hyperplasia does not correlate with the severity of glomerulonephritis in mouse strains with a Fas mutation^9^.

Cyclophosphamide treatment had more favorable results with respect to serum anti-dsDNA antibodies and the proportion of CD138, but ASC treatment also decreased the levels of anti-dsDNA antibodies significantly at 24, 30, 40 weeks of age and also decreased the proportion of CD138 significantly compared with the saline-treatment in female C3.MRL-Fas^lpr^/J mice. Decreases in CD138+ cells may lead to reduced anti-dsDNA antibody production, because CD138 is expressed in B cell precursors and plasma cells that are responsible for autoantibody production^24^,^25^. It was reported that the levels of circulating soluble CD138, the expression of CD138 mRNA in peripheral mononuclear cells, and the numbers of plasma cells increased in patients with active SLE compared with normal controls^26^. Furthermore, both cyclophosphamide and ASC treatment decreased the ratio of Th1/Th2 significantly compared with saline treatment. A previous study on the analysis of the Th1/Th2 balance in peripheral T helper cells revealed a strong predominance of Th1 among SLE patients with class IV lupus nephritis^27^. It was also reported that the serum levels of IFN-γ, TNF-α, and IL-12 were significantly higher in SLE patients than in healthy controls, as well as the Th1/Th2 (IL-12/IL-10, IL-12/IL-4, IFN-γ/IL-10, IFN-γ/IL-4, TNF-α/IL-10, and TNF-α/IL-4) ratios^28^.

Initiation and development of lupus nephritis and autoimmunity are dependent on complex multigenic interactions^9^. In the current study, the concentrations of urine protein and BUN and the UP/C ratio in the C3H background fas mutation mice were near the reference range and were much lower than in the MRL/MPJ background fas mutation mice. In histopathological examination of the kidney, MRL/lpr mice showed severe glomerulonephritis and severe infiltration of inflammatory cells in the interstitium and surrounding vessels^23^, but almost all C3.MRL-*Fas*^*lpr*^/J mice showed relatively benign lesions and only mild infiltration of inflammatory cells. Nevertheless, a few mice with mild-to-moderate or moderate glomerulonephritis were present only in the saline-treated control group and the fluorescence intensity of C3 deposition in all treatment groups was significantly lower than in the saline-treated control group.

MRL/lpr mice develop unique spontaneous skin inflammation like the cutaneous lesions of human lupus dermatitis; approximately 40% of saline-treated MRL/lpr mice showed scabs and hair loss on the dorsum of their neck at 24 weeks of age^23^. In contrast, all C3.MRL-*Fas*^*lpr*^/J mice in this study experienced no such skin lesions on gross and microscopic examination, even at 40 weeks of age.

The pathogenesis of SLE is affected by both genetic factors and epigenetic modifications, including DNA methylations, histone post-translational modifications and miRNAs^3^. miRNAs play a key role in the activation of innate immunity and the regulation of adaptive immune responses^29^. In this study, we intended to examine whether immunosuppressant or ASC treatment can modify disease-associated miRNAs. Thus, we compared the expression levels of the common lupus disease-associated miRNAs between the different treatment groups. The expression levels of miR-31-5p, miR-96-5p, miR-182-5p, miR-183-5p, and miR-379-5p were significantly higher, while those of miR150-5p were significantly lower in C3.MRL-*Fas*^*lpr*^/J mice (C group) than in C3H mice (N group).

Cyclophosphamide treatment led to significantly lower miR-96-5p, miR-182-5p, and miR-379-5p expression and significantly higher miR-150-5p expression relative to the saline-treated C3.MRL-*Fas*^*lpr*^/J control group. ASC treatment also led to significantly lower miR-96-5p and miR-182-5p expression compared with the saline-treated C3.MRL-*Fas*^*lpr*^/J control group. Thus, miRNAs have therapeutic implications, as well as being important in the pathogenesis of SLE. There was strong correlation between miR-96-p5 and the SLE parameters of miR-182-5p, spleen weight, lymph node weight, spleen weight/body weight, lymph node weight/body weight, glomerular C3 deposition, anti-dsDNA antibody levels (at 40 weeks of age), percentage of CD138+ cells, percentage of T-bet+ of CD4+ CD25+ cells, Th1/Th2, and percentage of CD4+ CD25+ Foxp3+ cells. There was also strong correlation between miR-182-p5 and some SLE parameters.

miR-182 and miR-96 target the transcription factor Foxo1/3a, and microphthalmia-associated transcript factor (MITF)^30^,^31^,^32^, are important in T and B cell homeostasis and tolerance, respectively^31^,^33^,^34^,^35^. The upregulation of miR-96 and miR-182 may downregulate Foxo1/3a^30^,^31^, leading to the breakdown of T cell tolerance^34^,^35^. Furthermore, there may be enhanced autoinflammation, and decreased expression of MITF^31^, which leads to enhanced spontaneous B cell activation and autoantibody production^33^. A decrease in Foxo1 transcript levels in peripheral blood mononuclear cells from SLE patients was also reported^36^. Thus, a decrease in miR-96-5p and miR-182-5p expression by ASC or cyclophosphamide treatment might result in reducing the breakdown of T cell tolerance and decreasing the production of autoantibodies, which are thought to be involved in the therapeutic mechanism.

In the results of the splenocytes from the MRL/lpr mice, ASC treatment did not change the miR expression significantly, but cyclophosphamide treatment decreased the expression of miR-31-5p, miR-96-5p, miR-127-3p, miR-182-5p, miR-183-5p, and miR-379-5p significantly compared with the saline-treatment. In the study using the MRL/lpr mice, the proportion of CD138 cells was significantly lower in the cyclophosphamide treatment group than in the saline-treatment group, but no significant difference in the proportion of CD138 cells was found in the ASC treatment group when compared with the saline-treatment group^23^.

We explored the possibility of osteogenic differentiation of systemic infused ASC. Systemic infusion of ASC did not alter the trabecular integrity in the mild SLE model or in the previous severe SLE model^23^. Treatment with cyclophosphamide led to a significant reduction in bone volume and trabecular number and an increase in bone spacing in the trabecular bone both MRL/lpr mice^23^ and C3.MRL-*Fas*^*lpr*^/J mice. Cyclophosphamide treatment results in inhibition of bone formation and reduced estrogen production, inferring that it can induce osteoporosis^37^.

Regarding biodistribution, little evidence of cell fluorescence was found in lungs in the previous study using MRL/lpr mice, but many ASCs labeled with the CM-DiI red fluorescent tracker dye were found in the lungs of the C3.MRL-*Fas*^*lpr*^/J mice.

When hASCs labeled with CM-DiI red fluorescent tracker dye were administered intravenously to NZB/W F1 mice, the fluorescent signal from cells in the lung was very strong at 6 weeks of age (before disease onset). In contrast, at an advanced stage of the disease, little evidence of fluorescent-labeled cells in the lung was obtained in NZB/W F1 mice in the previous study^22^. Therefore, we assume that the number of fluorescent-labeled cells in the lung might be dependent on the health status of the mouse and the stage and severity of the disease.

Taken together, ASC transplantation reduced anti-dsDNA antibody levels, glomerular C3 deposition and CD138 proportion significantly, without trabecular damage. A decrease in miR-96-5p and miR-182-5p expression as well as a decrease in the CD138 proportion and Th1/Th2 ratio might be involved in the mechanisms of therapeutic effects of ASCs in C3.MRL-*Fas*^*lpr*^/J mice with the SLE phenotype. Genetic background affects not only the onset and severity of systemic autoimmune symptoms, but also the response to therapy; C3.MRL-*Fas*^*lpr*^/J mice and MRL/lpr mice have a similar, but not identical, response to ASC therapy. Further studies are needed to clarify the mechanism involved in miRNA expression and changes after various treatments.

## Materials and Methods

### Human ASC preparation

Human ASCs were prepared from surplus frozen, banked stem cells (K-STEMCELL, Seoul, Korea), as described previously^38^; hASCs were obtained by simple liposuction from abdominal subcutaneous fat after the donor had provided informed consent. Adipose tissues were digested with 4 ml RTase (K-Stemcell) per 1 g fat under gentle agitation for 60 minutes at 37 °C and were filtered through a 100-μm nylon sieve, followed by centrifugation at 470 × g for 5 min. The pellet was resuspended in RCME (hASC attachment medium, K-Stemcell) containing 10% fetal bovine serum (FBS, Gibco BRL, Rockville, MD, USA) and centrifuged at 470 × g for 5 min. The supernatant was discarded, and the cell pellet was collected. The cell fraction was cultured overnight at 37 °C at 5% CO_2_ in RCME containing 10% FBS. After 24 hours, non-adherent cells were removed by washing with Dulbecco’s phosphate buffered saline (DPBS, WELGENE, Daegu, Korea). The cell medium was then changed to RKCM (hASC culture medium, K-Stemcell) containing 5% FBS. The cells were maintained for 4 or 5 days until confluent (passage 0). When the cells reached 90% confluency, they were detached using TryPLE express (Gibco BRL) and subculture-expanded in RKCM until passage 3. Anonymized hASCs from one donor were used in this study under an institutional review board (IRB)-approved exemption (Samsung Medical Center IRB, SMC 2014-02-070).

### Experimental animals

Breeding pairs of C3.MRL-*Fas*^*lpr*^/J (stock number #000480) and background-matched control C3H/HeJ mice (stock number #000659) were purchased from the Jackson Laboratory (Bar Harbor, ME, USA). This study was reviewed and approved by the Institutional Animal Care and Use Committee of Samsung Biomedical Research Institute. Samsung Biomedical Research Institute is accredited by the Association for the Assessment and Accreditation of Laboratory Animal Care International and abides by the guidelines of the Institute of Laboratory Animal Resources.

### Experimental groups and treatment protocol

For the *in vivo* study, the experimental groups consisted of a control group (C group, C3.MRL-*Fas*^*lpr*^/J, n = 20), a cyclophosphamide-treated group (Y group, C3.MRL-*Fas*^*lpr*^/J, n = 20), an ASC-treated group (H group, C3.MRL-*Fas*^*lpr*^/J, n = 20 per group), and a normal group (N group, C3H/HeJ, n = 15). All mice used in this experiment were female.

Mice in the C and N groups were infused with 150 μL of saline every two weeks from 5 weeks until 39 weeks of age, and each mouse in the H group was intravenously administered 10^6^ ASCs/150 μL of saline every two weeks from 5 weeks until 39 weeks of age (a total of 18 times). The Y group was injected intraperitoneally with 20 mg/kg of cyclophosphamide every week from five weeks until 39 weeks of age. From 5 mice per group, blood and tissue samples were obtained at 24 weeks of age. Blood and tissue samples were collected when the remaining mice were 40 weeks old.

### Determination of blood urea nitrogen (BUN), serum creatinine and anti-dsDNA antibodies

BUN and serum creatinine were determined with a DRI-CHEM 3000 Colorimetric analyzer (Fujifilm, Tokyo, Japan). Anti-dsDNA antibodies were measured in sera collected at 5, 12, 18, 24, 30 and 40 weeks of age, as previously described^21^; briefly, anti-dsDNA antibody concentrations were measured using a mouse Anti-dsDNA ELISA Kit (Shibayagi Co. Ltd., Ishihara, Shibukawa, Japan).

### Determination of proteinuria

Fresh urine was collected via abdominal massage. Urine protein was measured using the Coomassie Brilliant Blue method as previously described^22^. Urine creatinine was measured using a DRI-CHEM 3000 Colorimetric analyzer (Fujifilm, Tokyo, Japan) by diluting the urine in deionized water at a ratio of 1:100.

### Determination of the proportion of T regulatory cells and CD138 cells by flow cytometry

Splenocytes were collected from each mouse and the proportion of each T helper cell subset was analyzed: Th1 cells (CD4+ CD25+ T-bet+), Th2 cells (CD4+ CD25+ GATA-3+), Th17 cells (CD4+ CD25+ ROR-γt+), and Treg cells (CD4+ CD25+ Foxp3+). For analysis of the proportion of the T helper subsets, splenocytes were stained with antibodies to CD4 and CD25 (FITC rat anti-mouse CD4 and APC rat anti-mouse CD25; BD Biosciences, San Jose, CA, USA). Cells were washed, fixed, and intracellular staining was performed according to the manufacturer’s protocol for the Foxp3/transcription factor staining buffer set (eBioscience, San Diego, CA, USA) and intracellular antibodies (PE rat anti-mouse Foxp3; BD Biosciences, PE anti-RORγt, PE anti-T-bet, and PE anti-GATA-3; eBioscience). Fluorescence signals from 10,000 cells were counted, and flow cytometry analysis was carried out using a FACSAria flow cytometer (BD Biosciences). The proportion of CD138-positive cells was also determined (PE rat anti-mouse CD138, BD Biosciences).

### MicroRNA isolation and quantitative real-time PCR analysis in the spleen

Total RNA containing microRNA (miRNA) was isolated from whole splenocytes using the miRNeasy Mini Kit (Qiagen). cDNA was generated using the miScript II RT Kit (QIAGEN) and qPCR was performed using the miScript SYBR Green PCR Kit (QIAGEN) with the following miScript primer assays (QIAGEN): mmu_miR-17-5p, mmu_miR-18a-5p, mmu_miR-31-5p, mmu_miR-96-5p, mmu_miR-101a-3p, mmu_miR-127-3p, mmu_miR-146a-5p, mmu_miR-150-5p, mmu_miR-155-5p, mmu_miR-182-5p, mmu_miR-183-5p, mmu_miR-379-5p, RNU6-2, and SNORD96A. qPCR was performed according to the standard protocol provided by the manufacturer, and miRNA expression levels were normalized to the endogenous small RNA controls, RNU6-2 and SNORD96A (average CT) and calculated using the 2^−ΔΔCT^ method.

The spleen samples from MRL/lpr mice in the previous study (23) were also analyzed; spleen samples from the control group (C group, MRL/lpr, n = 12), a cyclophosphamide-treated group (Y group, MRL/lpr, n = 13), the ASC-treated groups (E and L groups, MRL/lpr, n = 12 and n = 13, respectively), the CTLA4Ig-ASC-treated group (CT group, MRL/lpr, n = 13), and the normal group (N group, MRL/MPJ, n = 9) were included. In the previous study, each group was treated as follows: each mouse in the C and N groups was infused with 150 μL of saline every two weeks from 5 weeks until 23 weeks of age. Each mouse in the E and CT groups was intravenously administered 10^6^ ASCs and CTLA4Ig-ASCs/150 μL of saline, respectively, every two weeks from 5 weeks until 23 weeks of age (a total of 10 times). Each mouse in the L group was intravenously administered 10^6^ ASCs/150 μL of saline every two weeks from 15 weeks until 23 weeks of age (a total of five times). The Y group was injected intraperitoneally with 20 mg/kg of cyclophosphamide every week from 5 weeks until 23 weeks of age^23^.

### ELISA of multiple cytokine levels in sera

Serum samples from all mice were assayed using a multiplex cytokine ELISA kit for GM-CSF, TNF-α, IFN-γ, IL-1α, IL-1β, IL-2, IL-4, IL-6, IL-10, IL-12p70, IL-15, and IL-17 (Millipore, Bedford, MA, USA).

### Histological analysis

Hematoxylin and eosin (H&E), Periodic acid-Schiff (PAS), and Masson’s trichrome staining of the kidney were conducted as previously described^21^.

### Immunofluorescence

Fresh kidney tissue was embedded in Optimum Cutting Temperature compound and frozen in 2-methyl-butane slush. Slides were incubated with FITC-conjugated goat anti-mouse IgG (Millipore) or FITC-conjugated goat anti-mouse C3 (West Chester, PA, USA), as previously described^21^. The slides were then mounted with mounting medium containing DAPI (Vector laboratories, Southfield, MI, USA), and examined with a laser scanning confocal microscope (LSM 700, Carl Zeiss, Jena, Germany). The intensity of fluorescence was graded on a scale of 0 (none) to 4 (great fluorescence intensity), and scoring was performed in a blinded manner.

### Ex vivo micro-computed tomography (micro-CT) imaging and analysis of the distal femoral metaphysis

Mice were sacrificed and the femurs were fixed in 4% formalin for micro-CT imaging, which was performed using a micro-CT scanner (Inveon Preclinical CT, Siemens Healthcare, Hoffman Estates, IL) at 40-μm slice thickness, with an exposure time of 600 msec, a photon energy of 70 keV and a current of 400 μA. The projection images were reconstructed into a three-dimensional image using IRW software (Siemens Healthcare). The bone parameters (bone volume/total volume, bone surface area/bone volume, trabecular thickness, trabecular number, and trabecular spacing) were calculated from 2.5 × 0.5 × 0.5 mm^3^ of distal femoral bone using Siemens Inveon Software.

### Confocal microscopy examination of CM-DiI-labeled ASCs

Three mice from the H group were administered ASCs labeled with a conjugated red fluorophore, Cell Tracker CM-DiI, to permit identification in histopathological sections. The tissue samples were harvested at the end of the study (40 weeks of age). The presence of CM-DiI-labeled cells was determined in various tissues, such as the spleen, lymph node, kidney, liver, lung and heart (by counterstaining with a mounting medium containing DAPI), with a laser scanning confocal microscope (LSM 700, Carl Zeiss, Jena, Germany).

### Statistical analysis

All results are expressed as the mean ± standard error of the mean (SEM). The results were analyzed and different groups were compared using a one-way analysis of variance (ANOVA) followed by *post hoc* Tukey’s multiple comparison tests. Differences with a confidence level of 95% or higher were considered statistically significant (*p* < 0.05). Significant (*p* < 0.05) differences from the control (C group) are indicated by an asterisk. Differences in cytokine levels among group were evaluated by the Kruskal-Wallis test. Pearson’s correlations were used to evaluate the relationships miRNA expression levels and other SLE parameters. Correlations were defined as weak (<0.3), moderate (0.3–0.7), or strong (>0.7). All statistical analyses were conducted using SPSS version 22.0 (SPSS Inc., Chicago, IL, USA).

## Additional Information

**How to cite this article**: Choi, E. W. *et al*. Mesenchymal stem cell transplantation can restore lupus disease-associated miRNA expression and Th1/Th2 ratios in a murine model of SLE. *Sci. Rep.* **6**, 38237; doi: 10.1038/srep38237 (2016).

**Publisher's note:** Springer Nature remains neutral with regard to jurisdictional claims in published maps and institutional affiliations.

## Supplementary Material

Supplementary Figures

## Figures and Tables

**Table 1:** Relationships between miR-96-5p expression level and other SLE parameters and between miR-182-5p expression level and other SLE parameters.

Parameter	miR96-5p		miR182-5p	
	Pearson’s correlation coefficient	P value (two-tailed)	Pearson’s correlation coefficient	P value (two-tailed)
miR96-5p	1	—	0.960	<0.001
miR182-5p	0.960	<0.001	1	—
Spleen weight	0.766	0.001	0.753	0.001
Lymph node weight	0.732	0.001	0.708	0.002
Spleen weight/body weight	0.772	<0.001	0.750	0.001
Lymph node weight/body weight	0.718	0.002	0.693	0.003
BUN	0.135	0.619	0.051	0.850
Glomerular IgG deposition	0.606	0.013	0.621	0.010
Glomerular C3 deposition	0.915	<0.001	0.904	<0.001
Urine protein, 40 weeks of age	0.319	0.229	0.273	0.306
Urine creatinine, 40 weeks of age	0.224	0.403	0.393	0.132
Urine protein/creatinine, 40 weeks of age	0.260	0.332	0.164	0.544
Anti-dsDNA antibody, 40 weeks of age	0.734	0.001	0.631	0.009
CD138	0.930	<0.001	0.918	<0.001
ROR-γt+	−0.151	0.699	−0.182	0.638
T-bet+	0.659	0.054	0.699	0.036
GATA3+	−0.062	0.874	−0.052	0.894
CD4+CD25+	−0.651	0.057	−0.642	0.062
ROR-γt+ of CD4+ CD25+	0.143	0.713	0.008	0.984
Tbet+ of CD4+ CD25+	0.844	0.004	0.815	0.007
GATA+ of CD4+ CD25+	−0.114	0.770	−0.127	0.745
CD4+ CD25+ Foxp3+	−0.829	0.006	−0.804	0.009
CD4+ CD25+ ROR-γt+	−0.284	0.458	−0.396	0.291
CD4+ CD25+T-bet+	0.056	0.887	0.038	0.923
CD4+CD25+GATA3+	−0.210	0.588	−0.219	0.571
Treg/Th17	−0.392	0.297	−0.249	0.518
Th1/Th2	0.829	0.006	0.841	0.004

Pearson’s correlations were used to evaluate the relationships miRNA expression levels and other SLE parameters. Correlations were defined as weak (<0.3), moderate (0.3–0.7), or strong (>0.7). SLE: systemic lupus erythematosus, BUN: blood urea nitrogen.
