# Supplementary material for: Mesenchymal stem cell transplantation can restore lupus disease-associated miRNA expression and Th1/Th2 ratios in a murine model of SLE
Source: Sci Rep. 2016 Dec 7;6:38237. doi: 10.1038/srep38237 (PMC5141468; doi:10.1038/srep38237)
Supplement: Supplementary Figures [file srep38237-s1.pdf]

Title:

Mesenchymal stem cell transplantation can restore lupus disease-associated miRNA expression and Th1/Th2 ratios in a murine model of SLE

Authors:

Eun Wha Choi[1,2,*], DVM, PhD, MinJae Lee[1], MS, Ji Woo Song[1], BS, Il Seob Shin[3], DVM, PhD, Sung Joo Kim[1,4,*], MD, PhD

[1]Laboratory Animal Research Center, Samsung Biomedical Research Institute, 81 Irwon-ro, Gangnam-gu, Seoul 135-710, Republic of Korea

[2] School of Medicine, Sungkyunkwan University, 81 Irwon-ro, Gangnam-gu, Seoul 135-710, Republic of Korea

[3] Biostar Stem Cell Research Center, K-STEMCELL, #2-305 IT Castle, 98 GasanDigital2-ro, Geumcheon-gu, Seoul 153-768, Republic of Korea

[4]Department of Surgery, Division of Transplantation, Samsung Medical Center, Sungkyunkwan University School of Medicine, 81 Irwon-ro, Gangnam-gu, Seoul 135-710, Republic of Korea

*Corresponding author E-mail: vet.cew@gmail.com

kmhyj111@gmail.com

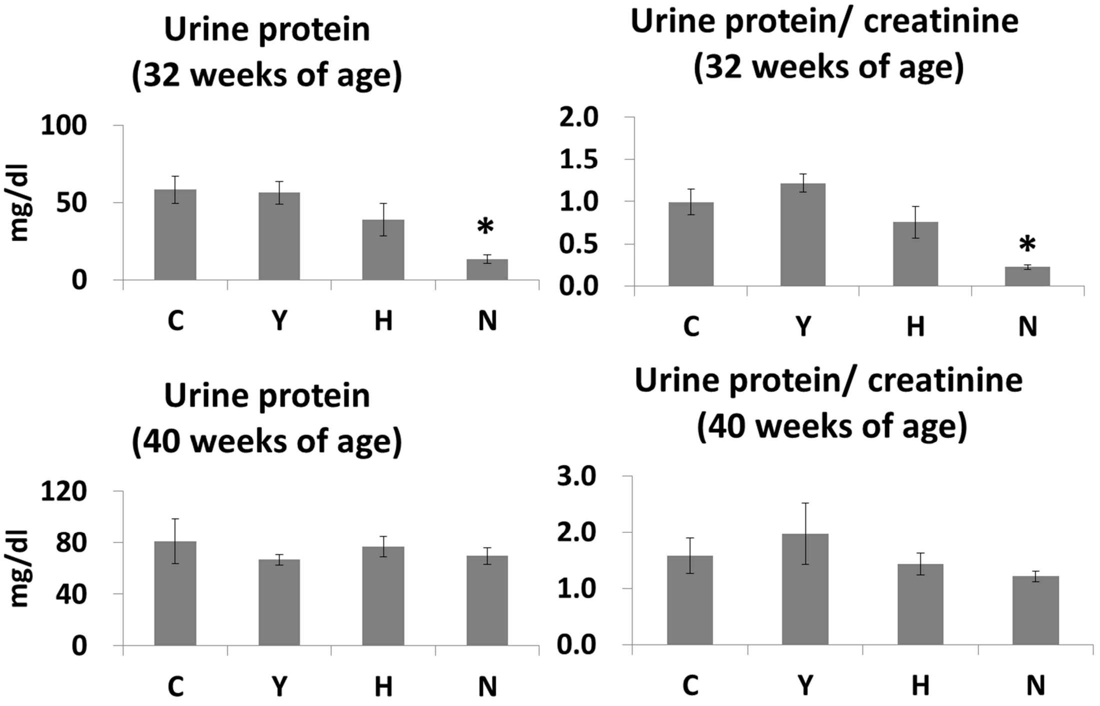

Supplementary Figure 1. Protein concentration and urine protein/urine creatinine ratio (UP/C) data in C3.MRL-Fas^{lpr}/J mice and background matched control C3H mice after treatment. Urine protein concentration and urine protein/urine creatinine ratio (UP/C) are presented. Data obtained from each group were compared using a one-way analysis of variance (ANOVA) followed by *post hoc* Tukey's multiple comparison tests. *: significant (p< 0.05) differences from the control (C) group are indicated by an asterisk. N: normal group (C3H), C: control group (C3.MRL-Fas^{lpr}/J), saline-, Y: cyclophosphamide-, H: ASC-treatment group.

A. Representative gating scheme

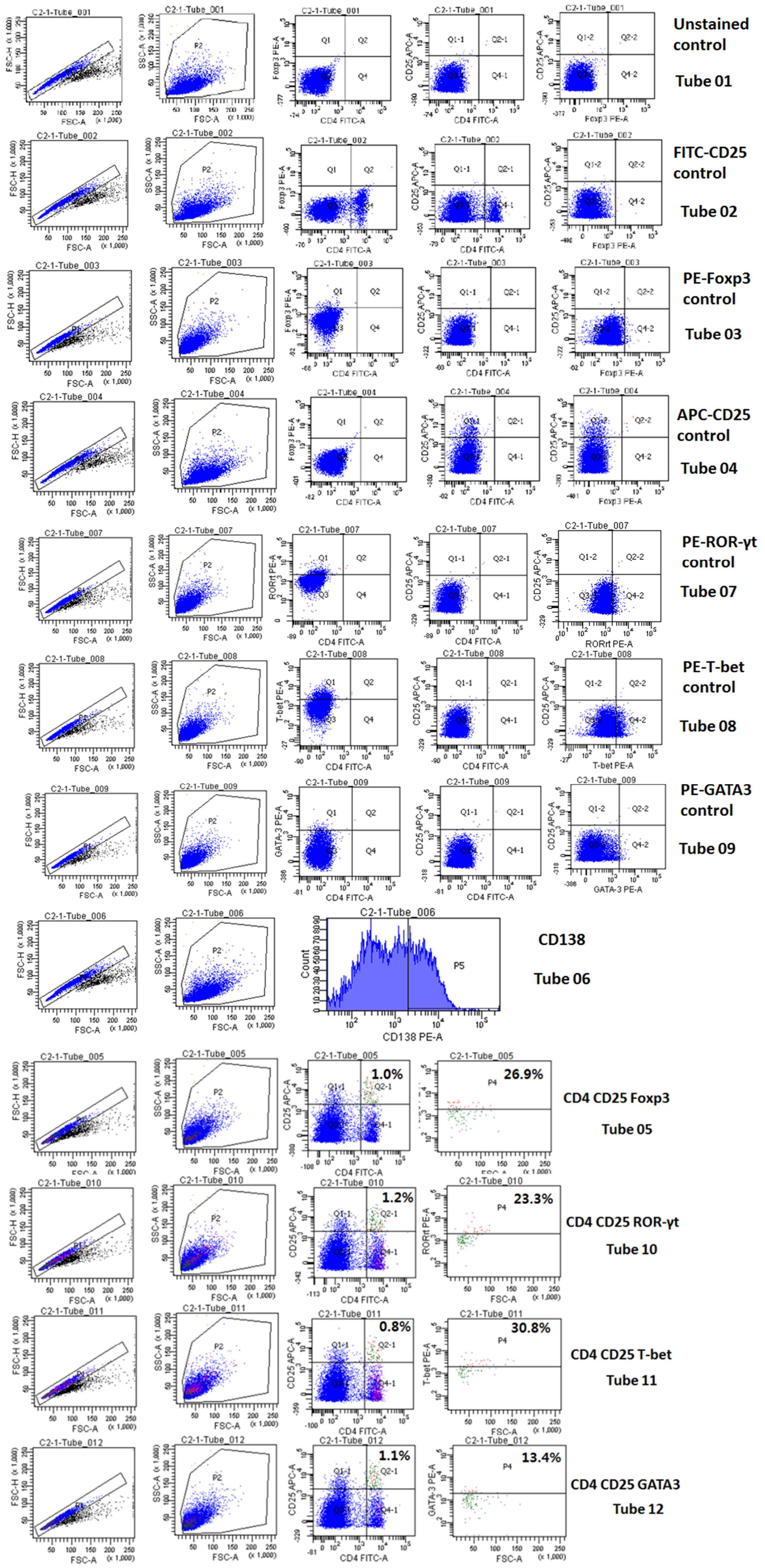

B. Representative multi-color flow cytometry gating analysis

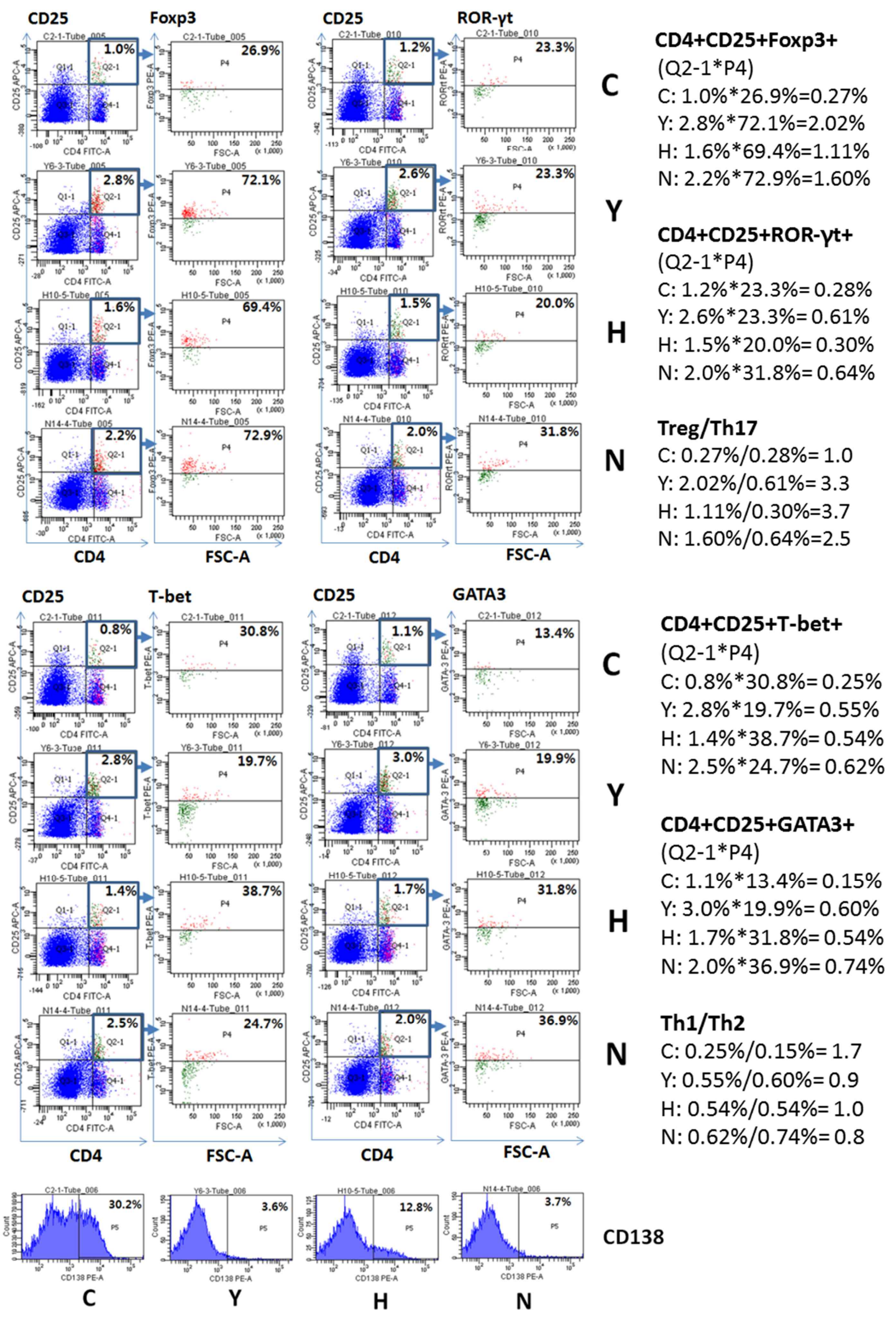

Supplementary Figure 2. Flow cytometric determination of the T helper cell subset (Th1, Th2, Th17, and Treg) and CD138 cells in the spleen. (A) A representative gating scheme and (B) representative multi-color flow cytometry gating analysis figures are presented. C: control group (C3.MRL-Fas^{lpr}/J mice), Y: cyclophosphamide-treatment group (C3.MRL-Fas^{lpr}/J mice), H: ASC-treatment group (C3.MRL-Fas^{lpr}/J mice) and N: normal group (Background-matched control C3H mice).

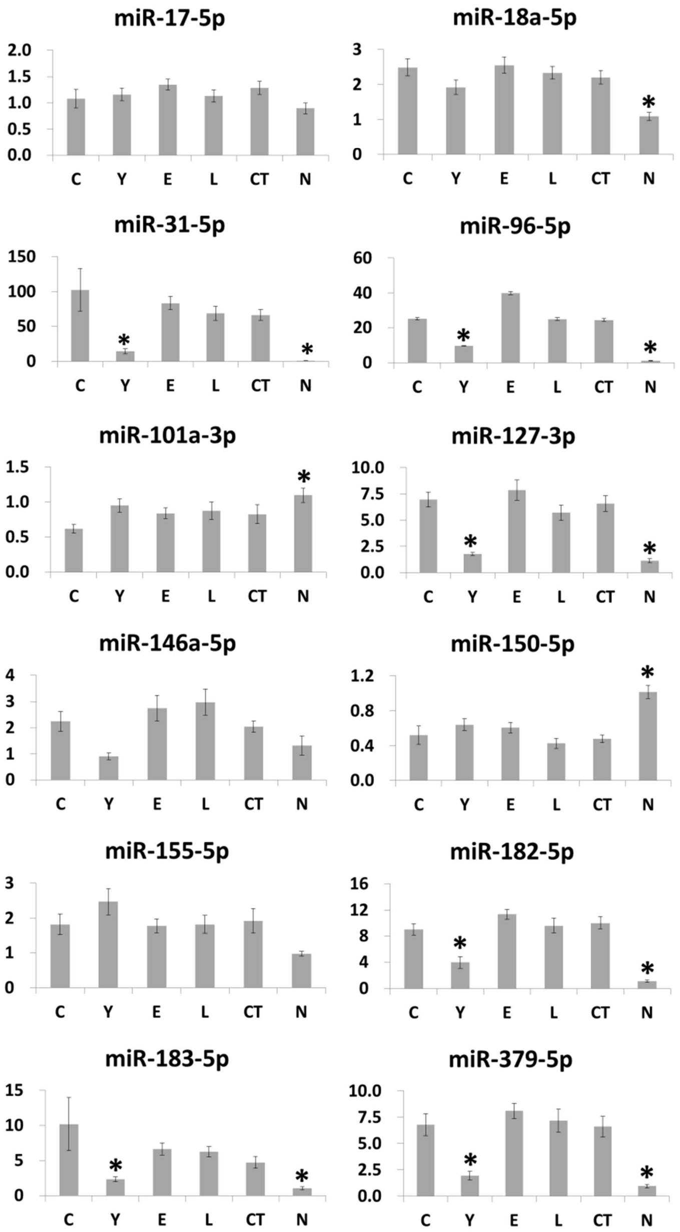

Supplementary Figure 3. Comparison of the expression levels of common lupus disease-associated miRNAs in spleens among the groups. The expression levels of common lupus disease-associated miRNAs in spleens. Data are normalized to those of RNU6-2 and SNORD96A (average CT) and are expressed as means (bars) ± SEM (error bars) of the relative amount compared to one sample of normal group. N: normal group (MRL/MPJ), C: control group (MRL/lpr), saline-, Y: cyclophosphamide-, E: ASC-early, L: ASC-late and CT: CTLA4Ig-ASC-treatment group. Data obtained from groups were analyzed using a one-way analysis of variance followed by *post hoc* Tukey's multiple comparison tests. *: significant ($p < 0.05$) differences from the control (C) group are indicated by an asterisk.

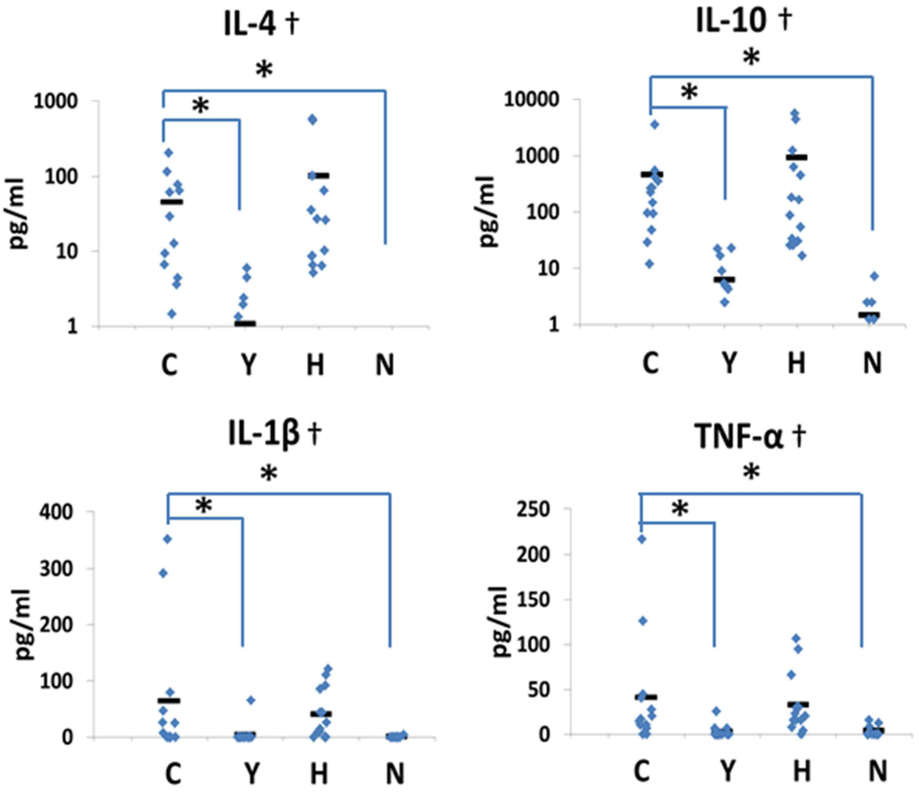

Supplementary Figure 4. Cytokine levels in sera from each experimental group. Data obtained from each group were compared using the Kruskal Wallis test (†: P< 0.05). *: Significant (p< 0.05) differences versus the C group are marked by asterisk (Mann-Whitney U test). C: control group (C3.MRL-Fas^{lpr}/J mice), Y: cyclophosphamide-treatment group (C3.MRL-Fas^{lpr}/J mice), H: ASC-treatment group (C3.MRL-Fas^{lpr}/J mice) and N: normal group (Background-matched control C3H mice).
